# Reprogramming SREBP1‐dependent lipogenesis and inflammation in high‐risk breast with licochalcone A: A novel path to cancer prevention

**DOI:** 10.1002/ijc.70226

**Published:** 2025-11-27

**Authors:** Atieh Hajirahimkhan, Elizabeth T. Bartom, Carolina H. Chung, Xingyu Guo, Kyli Berkley, Seyyedmohsen Hosseinibarkooie, Zahra Assadi, Shao Huan Samuel Weng, Raymond Moellering, Oukseub Lee, Ruohui Chen, Wonhwa Cho, Sriram Chandrasekaran, Susan E. Clare, Seema A. Khan

**Affiliations:** ^1^ Department of Surgery, Feinberg School of Medicine Northwestern University Chicago Illinois USA; ^2^ Robert H. Lurie Comprehensive Cancer Center Northwestern University Chicago Illinois USA; ^3^ Department of Biochemistry and Molecular Genetics Northwestern University Chicago Illinois USA; ^4^ Department of Preventive Medicine Northwestern University Chicago Illinois USA; ^5^ Department of Biomedical Engineering University of Michigan Ann Arbor Michigan USA; ^6^ Department of Chemistry University of Illinois Chicago Chicago Illinois USA; ^7^ Proteomics Platform, Office of Shared Research Facilities University of Chicago Chicago Illinois USA; ^8^ Department of Chemistry The University of Chicago Chicago Illinois USA

**Keywords:** anti‐inflammation, antiproliferation, breast cancer prevention, licochalcone a, SREBP

## Abstract

Anti‐estrogens have had a limited impact on breast cancer (BC) prevention. Novel agents with better tolerability, and efficacy beyond estrogen receptor (ER) positive BC are needed. We studied licochalcone A (LicA) for ER‐agnostic BC prevention. We demonstrated that LicA significantly reduced proliferation in seven human breast cell lines and suppressed ER+ and ER− xenograft tumors in mice. We confirmed these observations ex vivo in the contralateral unaffected breast (CUB) of women with unilateral sporadic BC, and BC cell lines using RNA sequencing, metabolism flux modeling, confirmatory NanoString nCounter metabolic pathway panel analysis in independent sets of specimens, proteomics, and western blots. We found that LicA targets sterol regulatory element binding protein 1 (SREBP1) with subsequent metabolic‐inflammatory changes, lowering spatiotemporally resolved cholesterol levels inside malignant cells to the levels in normal mammary cells. Mechanistically, in CUBs we observed that LicA downregulated PI3K‐AKT‐SREBP1‐dependent lipogenesis, NF‐kB‐dependent inflammation, and de novo nucleotide biosynthesis, stalling proliferation. Studies in cell lines showed suppression of PI3K and AKT phosphorylation, SREBP1 protein expression, and the SREBP1‐dependent enzymes such as ACAT2, ACLY, FASN, SCD, consistent with reduced NEDD8 required for SREBP1 stabilization. We found a significant reduction in NF‐kB expression, its nuclear translocation mediator karyopherin *β*1, and prostaglandin E2 synthesis. We demonstrated a reduction in PRPS1‐catalyzed de novo nucleotide biosynthesis, and downregulation of proliferative markers MKI67, RRM2, and the survival marker BCL2. LicA reduces pro‐tumorigenic aberrations in lipid homeostasis and inflammation through SREBP1. It is a promising non‐endocrine candidate for BC prevention. Future studies in immunocompetent BC prevention models are warranted.

## Graphical abstract

What's new?

Endocrine drugs repurposed from treatment protocols are the primary medications available for breast cancer (BC) prevention in at‐risk women. Adverse effects, however, significantly minimize uptake and prevention impact. Here, the authors investigated licochalcone A (LicA), a non‐endocrine anti‐inflammatory agent with reported hematologic and bone safety, as a potential alternative. Experiments show that LicA reduces human BC cell proliferation in vitro and suppresses ER+ and ER− xenograft tumors in mice. Similar effects were observed in breast microstructures from high‐risk women. Mechanistic studies reveal that LicA targets the sterol regulatory element binding protein (SREBP1)‐dependent metabolism, reducing pro‐tumorigenic aberrations. The findings warrant further investigation of LicA for BC prevention.

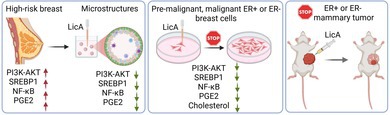

**Abbreviations**
ACAT2: Acyl‐CoA: cholesterol acyltransferase 2
ACLY: ATP citrate lyase
ADP: adenosine diphosphate
AI: aromatase inhibitor
AKT: protein kinase B
AMP: adenosine monophosphate
BC: breast cancer
BCL2: B cell lymphoma 2
CLS: crown‐like structures
CYP19A1: cytochrome P450 family 19 subfamily A member 1
CYP1B1: cytochrome P450 family 1 subfamily B member 1
ECM: extracellular matrix
EGFR: epidermal growth factor receptor
ER: estrogen receptor
FASN: fatty acid synthase
FBA: flux‐based analysis
GLS: generalized least square
IACUC: Institutional Animal Care and Use Committee
IPM: inner leaflet of the plasma membrane
KEAP1: kelch‐like ECH‐associated protein 1
KPNB1: karyopherin subunit beta‐1
LicA: licochalcone A
MKI67: marker of proliferation ki‐67
NAD(P)H: nicotinamide adenine dinucleotide (phosphate)
NEDD8: neural precursor cell expressed developmentally down‐regulated 8
NF‐kB: nuclear factor kappa‐light‐chain‐enhancer of activated B cells
NRF2: nuclear factor erythroid 2‐related factor 2
p‐AKT: phospho AKT
PGE2: prostaglandin E2
PI3K: phosphoinositide 3‐kinase
p‐PI3K: phospho PI3K
PPP: pentose phosphate pathway
PR: progesterone receptor
PRPS1: phosphoribosyl pyrophosphate synthase 1
R‐1‐P: ribose 1 phosphate
R‐5‐P: ribose 5 phosphate
RO5: rule of 5
ROS: reactive oxygen species
RRM2: ribonucleotide reductase M2
s.c.: subcutaneous
SCD: stearyl CoA desaturase
SDS‐PAGE: sodium dodecyl sulfate polyacrylamide gel electrophoresis
SERMs: selective estrogen receptor modulators
SREBF: sterol regulatory element factor
SREBP: sterol regulatory element binding protein

## 1 BACKGROUND

Current recommendations for BC prevention are based on estimates of risk in individual women^1^; for those at markedly increased risk due to high‐penetrance germline mutations, risk‐reducing bilateral mastectomy can be considered. However, most at‐risk women are at risk for other reasons, with risk estimates in the 1.5–5‐fold range. For them, risk‐reducing medications are recommended. At present, the only risk‐reducing drugs available for BC are anti‐estrogens: aromatase inhibitors (AIs) for postmenopausal women and selective estrogen receptor modulators for pre and postmenopausal women. These drugs can lower the risk of estrogen receptor positive (ER+) BC by 50–65%.^2^ However, of the estimated 10 million US women who could benefit from these medications more than 85% decline them, mainly due to their adverse effects: vasomotor symptoms, thrombogenesis/stroke, bone loss, and endometrial cancer.^2^, ^3^ In addition, these drugs do not prevent estrogen receptor negative (ER−) BC. One of the pathways abnormally active in atypical hyperplasia and ductal carcinoma in situ (DCIS) is PI3K‐AKT.^4^, ^5^ PI3K pathway inhibitors such as everolimus and alpelisib have been proposed for repurposing as ER‐ and ER+ BC prevention agents^6^, ^7^, ^8^; however, their side effects such as hyperglycemia are a major impediment.^9^ There have been attempts to repurpose widely used and well‐tolerated drugs such as metformin for BC risk reduction; however, clinical trial results have been disappointing.^10^, ^11^ Therefore, there is a clear need to have alternative agents with sufficient efficacy, minimal adverse effects, and greater acceptance.

Upregulated de novo fatty acid and cholesterol biosynthesis have an important role in BC development and progression through enhanced proliferation, inter and intracellular signaling, and enabling evasion from immune surveillance.^12^ The activity of the master regulator of lipogenesis, sterol regulatory element binding protein 1 (SREBP1) has been shown to have prognostic value in BC progression.^13^, ^14^ SREBP1 plays a major role in macrophage polarization and regulation of the inflammasome in the tissue microenvironment and forms a complex with NF‐kB that regulates the inflammatory response.^15^ Excess activation of SREBP1 upregulates the expression of most fatty acid biosynthesis enzymes and SREBP2‐dependent cholesterol biosynthesis. This subsequently promotes an inflammatory/oxidative microenvironment, provides excess cell membrane structural components necessary for proliferating cells, protects malignant cells from immune destruction, promotes cancer stem cell viability, and supplements cholesterol and its derivatives as precursors for estrogen biosynthesis.^16^ The role of dysregulated lipogenesis in promoting ER− BC is associated with the inflammatory cytokines secreted from immune cells recruited to the stroma consequent to excess free fatty acids and cholesterol in the microenvironment, and the dysregulation of metabolic hormones and growth factors.^16^, ^17^ The cancer‐promoting effects of inflammation^18^ can be tempered through downregulating SREBP1 and through the activation of NRF2 and suppression of NF‐kB‐dependent pathways.^15^, ^19^ This presents an opportunity for interventions that prevent malignant transformation in the breast. Importantly, such a strategy will not produce deleterious endocrine responses that are of concern with presently available risk reduction drugs.^19^

Our current research reveals that licochalcone A (LicA), a compound that is clinically studied for the relief of inflammatory skin conditions, is an excellent candidate as a non‐endocrine BC risk‐reducing drug with minimal toxicity and greater acceptance. It has antioxidant and anti‐inflammatory effects,^20^, ^21^, ^22^ and moderately inhibits aromatase (CYP19A1), the target of AIs used for BC prevention.^23^ In addition, LicA blocks estrogen genotoxic metabolism through the downregulation of CYP1B1, in vivo.^24^ LicA has also been reported to relieve conditions such as arthritis, mastitis, and acute lung injury by blocking inflammation, and appears to have osteoprotective effects.^25^, ^26^, ^27^ The anti‐inflammatory effects of LicA can be partly explained by its interaction with KEAP1 and its subsequent activation of NRF2 and suppression of NF‐kB,^20^, ^28^ its effect on IKK/NF‐kB signaling and translocation of p65 to the nucleus^29^, ^30^, ^31^ but it can also be one of the consequences of its effects on SREBP1.^15^ In the current study, we confirm our previous in vitro and in vivo antioxidant observations with LicA, in breast microstructures from women at high risk of BC. We also present novel findings regarding its effects on metabolic pathways involving PI3K‐AKT‐SREBP1 which we have elucidated in various breast cell lines, human ex vivo, and in vivo models using multiple orthogonal approaches. In addition, we show its antiproliferative effects resulting from changes in flux through the key metabolic pathways. Based on these results and existing literature, we posit that reducing lipogenesis and inflammation through suppressing SREBP1 can reduce the risk of BC and that LicA presents as an excellent candidate for BC prevention and is highly likely to be safe and well‐tolerated.

## 2 MATERIALS AND METHODS

### 2.1 Chemicals and materials

All chemicals and reagents were purchased from Sigma‐Aldrich (St. Louis, MO), unless otherwise indicated. The detailed list is included in the Supplementary Materials.

### 2.2 Cell culture

We grew pre‐malignant DCIS.COM (PRID: CVCL_5552) and malignant MCF‐7 (PRID: CVCL_0031) and MDA‐MB‐231 cells (PRID: CVCL_0062) (all obtained from ATCC) in RPMI‐1640 supplemented with 10% fetal bovine serum (FBS) and 1% penicillin/streptomycin (Invitrogen Thermo Fisher Scientific, Hanover Park, IL.). We also grew pre‐malignant DCIS.COM/ER+ PR+ cells obtained from Dr. Dean Edwards's laboratory (Baylor College of Medicine)^32^ in DMEM/F12 supplemented with 10% horse serum, 0.5% penicillin/streptomycin, and 1% HEPES. In addition, we acquired MCF‐7 cells overexpressing aromatase, MCF‐7aro (RRID: CVCL_9580), from Dr. Shiuan Chen's laboratory (City of Hope)^33^ and grew them in MEM supplemented with 10% FBS, 2 mM L‐glutamine, 1 mM sodium pyruvate, 1% nonessential amino acids, and 1 U/mL penicillin/streptomycin. BRCA defective malignant breast cell lines HCC‐1937 (RRID: CVCL_0290), and HCC‐3153 (RRID:CVCL_3377) were obtained from Dr. Gazdar's group (University of Texas Southwestern Medical Center) and were grown in RPMI‐1640 supplemented with 5% FBS and 1% penicillin/streptomycin. All human cell lines have been authenticated using STR profiling within the last 3 years. All experiments were performed with mycoplasma‐free cells.

### 2.3 Human breast microstructure preparation and treatment

We prepared breast microstructures from the unaffected contralateral breast of postmenopausal women who underwent bilateral mastectomy for unilateral BC, as described previously, with minor modifications.^34^ The tissues were obtained while fresh and diced to approximately 5 mm pieces before digesting at 37°C with 2% collagenase I in F12‐K nutrient mix (Kaighn's) medium, overnight. After the completion of digestion, the tissue was centrifuged at 250 × *g* for 5 min and the supernatant was removed. The pellet was washed with phosphate‐buffered saline and was mixed and cultured in MammoCult media supplemented with 0.2% heparin and 0.5% hydrocortisone. After 24 h incubation at 37°C the treatments were mixed with fresh MammoCult media and added to the microstructures.

### 2.4 RNA sequencing

Microstructures obtained from 6 subjects were incubated with LicA (5 μM) or DMSO. After 24 h they were washed with HBSS. Trizol was added to each pellet and the protocol of the Direct‐Zol RNA prep kit was used to extract RNA. The protocol included a step of DNAse I treatment for removing DNA contamination i the RNA preparations. RNA was eluted in nuclease‐free water, and quality assessment was performed using Agilent Bioanalyzer and Qubit. Libraries were made using the Roche KAPA Biosystems protocol (KAPA RNA Hyper Prep Kit Technical Data Sheet, KR1352—v4.17, Roche Corporate) and the quality was evaluated. Total RNA sequencing was performed with Illumina NovaSeq 6000 v1.5, 2 × 100 b, paired end with 40 million reading depth, followed by quality assessment using FASTQC, alignment to the reference genome hg38, raw data processing using STAR, and data analysis to define differential gene expression, as described previously.^35^, ^36^ Significantly (adj *p* <.05) modulated genes were further analyzed with Enrichr to define highly modulated pathways. The sample relationships were validated with NGSCheckMate as well. A more detailed protocol is provided in the Supplementary Materials. The sequencing coverage and quality statistics for each sample are summarized in the Supplementary Table S1.

### 2.5 NanoString nCounter™ metabolism pathway panel

We treated six additional subjects' breast microstructures and two cell lines MDA‐MB‐231 (ER−) and MCF‐7 (ER+) cells with LicA (5 and 10 μM) or DMSO for 24 h. After extracting RNA and conducting quality assessment we used the NanoString nCounter Metabolism Panel (NanoString Technologies Inc.) to quantify RNA expression from 749 genes (Table S2). Differentially expressed genes and pathway analysis were determined using the ROSALIND platform (ROSALIND, Inc.) with normalization, fold changes (≥1.5 fold: upregulated or ≤−1.5 fold: downregulated), and adjusted *p*‐value <.05 using the Benjamini‐Hochberg method. A detailed protocol is provided in the Supplementary Materials. The probes and counts for each sample are summarized in Supplementary Table S3.

### 2.6 Genome‐scale metabolic network modeling and flux‐based analysis (FBA)

We calculated the relative activity of reactions in breast microstructures of 6 women by interpreting gene expression data using the Recon1 human metabolic model as outlined previously.^37^, ^38^ We then identified a metabolic flux state that is most consistent with gene expression data in control and LicA‐treated samples. This was achieved by maximizing the activity of reactions that are associated with upregulated genes and minimizing flux through reactions that are downregulated in a condition, while simultaneously satisfying the stoichiometric and thermodynamic constraints embedded in the model using linear optimization.^37^, ^38^ The glucose, fatty acid, and glutamine levels in the simulations were adjusted based on the growth media used for culturing the high‐risk women's breast microstructures. All p‐values were corrected for multiple comparisons.

### 2.7 Proliferation assay

We seeded pre‐malignant DCIS.COM (ER‐ PR‐) and DCIS.COM/ER+ PR+ cell lines as well as malignant MCF‐7 (ER+ PR+), MDA‐MB‐231 (ER‐ PR‐), MCF‐7aro (ER+ PR+ overexpressing CYP19A1), HCC‐1937 (ER‐ PR‐, BRCA1‐mutated), and HCC‐3153 (ER− PR−, BRCA1‐mutated) cells with the density of 2.5 × 10^3^ cells/well in their appropriate media in 96‐well plates. We placed the plate in IncuCyte instrument for continued live cell imaging every 6 h. When cells reached 30% confluency, eight different concentrations of LicA ranging from 350 nM to 40 μM or respective DMSO control were added to the designated wells. A detailed experimental procedure is provided in the Supplementary Materials. Data were analyzed using Zoom software to quantify the images and were plotted as mean ± SEM of at least two independent measurements.

### 2.8 Proteome integral solubility alterations (PISA) proteomics

MCF‐7 and MDA‐MB‐231 cells were treated with LicA (10 μM) for 24 h. We used the sample preparation and PISA protocol as described previously,^39^ with minor modifications. The cells were detached and centrifuged. The cell pellets were reconstituted in PBS containing protease and phosphatase inhibitors. Thermal denaturation was performed with a steady temperature at 40, 42, 45, 50, 55, 60, 63, and 65°C followed by freeze–thaw cycles and BCA assay (Thermo Fisher Scientific) to quantify the proteins. Using S‐Trap column, proteins were captured and washed before conducting LC–MS/MS. A detailed procedure is provided in the Supplementary Materials. Proteome Discoverer was used for data analysis.^39^ Statistical analysis was performed using Python. The differentially expressed proteins were also analyzed by Enrichr to obtain significantly stabilized and destabilized pathways.

### 2.9 Western blot

The Western blot analysis was performed as described before.^20^ After 48 h of treatment, the cells were lysed and the supernatants were collected. Proteins were quantified using a BCA assay kit, and loaded on 4%–12% sodium dodecyl sulfate polyacrylamide gel electrophoresis (SDS‐PAGE), followed by transfer to a polyvinylidene difluoride membrane, blocking with 5% (w/v) skim milk in TBS‐T, and incubations with the primary and secondary antibodies as detailed in the Supplementary Materials. The membranes were detected using Clarity™ Western ECL Blotting Substrates (Bio‐Rad, Hercules, CA, USA) and a Bio‐Rad imager. Quantification of the images was performed using the Image J software.

### 2.10 In situ quantitative imaging of cellular cholesterol

Spatiotemporally resolved in situ quantification of cholesterol in the inner leaflet of the plasma membrane (IPM) of mammalian cells was performed using a ratiometric cholesterol sensor, WCR‐*e*Osh4 as described previously.^40^, ^41^ WCR‐*e*Osh4 was prepared and calibrated using giant unilamellar vesicles as described previously.^40^ WCR‐*e*Osh4 was microinjected into cells, and the cholesterol concentration in the IPM was determined using in‐house programs written in MATLAB as described.^40^ The three‐dimensional display of the local cholesterol concentration profile was calculated using the Surf function in MATLAB.

### 2.11 In vivo studies

Female ovary‐intact athymic nude mice (6 weeks old) were purchased from Jackson Laboratory. After a week of acclimation, they were subcutaneously inoculated with either 1 million MCF‐7 (12 animals) or MDA‐MB‐231 cells (18 animals). When tumors reached 0.8 cm in diameter, treatment with LicA (80 mg/kg/day) or vehicle (4% DMSO, 6% EtOH, 45% water, 45% PEG‐400) began and continued for 28 days. Animals were weighed weekly, and tumor sizes were measured twice weekly using digital calipers. Tumor volume was calculated with the formula [Tumor volume = (*A* ^2^) × *B*/2], where “*A*” is the smaller diameter and “*B*” is the larger. Tumor growth in LicA‐treated animals was compared to vehicle controls. At the end of treatment, tumors were harvested, weighed, and preserved. A more detailed procedure is included in the Supplementary Materials. Animals meeting humane endpoint criteria as per IACUC protocol were sacrificed before treatment completion.

### 2.12 Statistical analysis

The regular ordinary least squares regression (e.g., simple linear regression) does not consider heterogeneity across groups or time. Therefore, we fit a linear model using generalized least squares to compare the tumor size trend over time between groups using LicA and vehicle for MDA‐MB‐231 and MCF‐7 xenografts. We also compared the marginal tumor size between groups using LicA and vehicle for MDA‐MB‐231 and MCF‐7 xenografts through least‐squares means. We compared different variance–covariance structures, through ANOVA and selected the best fitted model with the smallest AIC.

## 3 RESULTS

### 3.1 Licochalcone A alters gene expression and pathway activity in breast microstructures from high‐risk postmenopausal women

**FIGURE 1:**
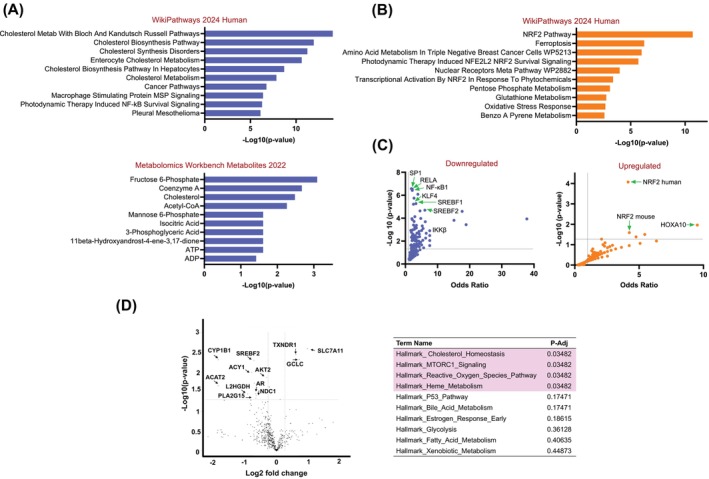
Transcriptomics analysis in postmenopausal high‐risk women's breast microstructures treated with LicA. Using RNA sequencing, LicA in the first set of 6 subjects' specimens (A) downregulates cholesterol and lipid metabolism, (B) upregulates antioxidant pathways, (C) downregulates transcription factors associated with inflammation, proliferation, lipid and cholesterol metabolism, and upregulates transcription factors associated with antioxidant response: Data derived from TRRUST_Transcription_Factors_2019 gene set, (D) shares significant similarities in downregulated and upregulated transcriptomic signatures with statins: Data derived from drug matrix geneset. (E) NanoString nCounter™ metabolism panel analysis of six additional postmenopausal high‐risk women's breast microstructures. LicA downregulates genes associated with lipid and cholesterol metabolism and estrogen carcinogenic metabolism and upregulates antioxidant response genes.

LicA alters the transcriptional profile of women's breast tissues compared to DMSO. Differential gene expression analysis revealed 2341 downregulated genes (adj *p* <.05), with 381 having log2FC <−1, including *HMGCR*, *MVD*, *MVK*, *SQLE*, *ACAT2*, *SREBF1*, *SREBF2*, *INSIG1*, *LSS*, *PTGST*, *RELA*, *ALDH1A3*, *SERPINB2*, *Wnt*, and *CYP1B1*. In contrast, 1462 genes were upregulated (adj *p* <.05), with 538 having log2FC >1, such as *NQO1*, *NQO2*, *HMOX1*, *GST*s, *TXN*, *TXNRD1*, *SLC*s, *G6PD*, and *GCLC*. Pathway enrichment analysis showed that downregulated processes include lipid and cholesterol metabolism, PI3K–AKT signaling, and inflammation (Figures 1A, S1A), with cholesterol and acetyl‐CoA predicted as decreased metabolites. Upregulated pathways involved NRF2‐dependent antioxidant processes, the pentose phosphate pathway (PPP), and ferroptosis (Figures 1B, S1B). Transcription factor analysis (Figure 1C) indicated *NRF2* was significantly upregulated in LicA‐treated samples, while proliferative factors *SP1*, *KLF4*, inflammation transcription factor *NF‐kB* and its subunit *RELA*, and the lipogenic transcription factors sterol regulatory element binding factor (*SREBF*) *1* and *SREBF2* were downregulated. (Figure 1C).

*Validation of sequencing results* , using the NanoString metabolism panel consisting of 749 metabolic genes demonstrated (Figure 1D) significant (*p* <.05) downregulation of up to 3.5‐fold change in *ACAT2*, *CYP1B1*, *L2HGDH*, *ACY1*, *SREBF2*, *NRF1*, *AR*, *PLA2G15*, *AKT2*, and *NDC1* expression and significant (*p* <.05) upregulation of up to 1.9‐fold change in *SLC7A11*, *GCLC*, *TXNRD1* expression (Figure 1D). The analysis also showed that pathways associated with cholesterol homeostasis, mTORC1 signaling, reactive oxygen species metabolism, and heme metabolism were significantly modulated (adj *p* <.03) by LicA (Figure 1D).

### 3.2 Licochalcone A changes flux through metabolic pathways to facilitate support for antioxidant effects, reduced lipogenesis, and reduced proliferation

**FIGURE 2:**
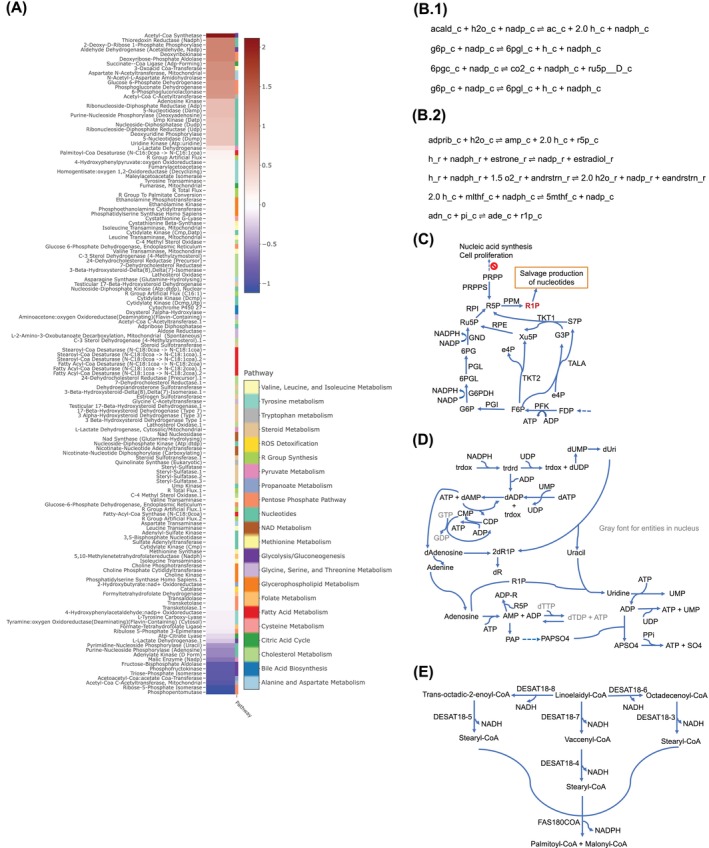
Genome‐scale metabolic flux balance analysis in six postmenopausal high risk women's breast microstructures. (A) Metabolic flux heat map identifies significant flux (*p*‐value <.01) through antioxidant pathways, lipid and cholesterol biosynthesis and metabolism. Magnitude of flux difference between treatment and control are shown. (B) Metabolic flux in the representative pathways is (B.1) toward the right direction and (B.2) toward the left direction to enhance NAD(P)H generation. (C) Metabolic flux through pentose phosphate shunt is in the direction generating NAD(P)H without enhancing nucleotide biosynthesis. (D) Nucleotides essential for bioenergetics and kinetics of the metabolic reactions are salvaged. (E) Flux through fatty acid elongation drives NAD(P)H generation and formation of saturated fatty acids.

In the first 6 high‐risk women's tissue microstructures genome‐scale metabolic modeling (Figure 2)^42^ showed that flux through 191 reactions was significantly (*p* <.05) modulated as a function of exposure to LicA. Some of the top modulated metabolic reactions are key steps in glycolysis/gluconeogenesis, nucleotide biosynthesis and catabolism, PPP, pyruvate metabolism, cholesterol metabolism, fatty acid elongation, and steroid metabolism (Figure 2A). Interestingly, when the results were further explored at the metabolic reaction level using the BIGG database,^43^ the direction of the flux for many reactions was in favor of generating excess NAD(P)H and creating a reducing/antioxidant environment (Figure 2B). Examples of these reactions were aldehyde dehydrogenase in glycolysis/gluconeogenesis, glucose‐6‐phosphate dehydrogenase and phosphogluconate dehydrogenase in PPP, 17‐beta hydroxysteroid dehydrogenase in steroid metabolism, and methylene tetrahydrofolate reductase in folate metabolism (Figure 2B). Flux through the NADPH‐generating oxidative branch of PPP was significantly increased (Figure 2C). Flux through the non‐oxidative branch of PPP was toward the generation of ribose 5 phosphate (R5P) (Figure 2C). However, flux through PRPPS, which is responsible for de novo nucleotide biosynthesis, was not in favor of PRPP generation; rather the production of R5P was favored (Figure 2C). This was consistent with the subsequent enhanced flux through PPM‐catalyzed reactions involved in nucleotide catabolism and salvage production of nucleotides. Thus, R5P is consumed to generate R1P, supporting efficient recycling of nucleotides for DNA repair or energetics, instead of R5P formation essential for de novo nucleotide biosynthesis and cell proliferation (Figure 2C).

*Overall nucleotide flux* (Figure 2D) offered further confirmation of the above findings. We observed the dominance of the salvage nucleotide biosynthesis process and its connection to antioxidant reactions such as NADPH‐dependent formation of reduced thioredoxin (Figure 2D). This was further made evident when we observed that the formation of ADP‐ribose from AMP and R5P is favored over the formation of R5P, leading to reduced proliferation (Figure 2B,D). Similarly, we observed that flux through several reactions in cholesterol metabolism (Figure S2) such as 4‐methyl zymosterol, 24‐dehydrocholesterol reductase, 7‐dehydrocholesterol reductase, lathosterol oxidase, cholesterol efflux, and several cholesterol transporters was in a direction to generate less cholesterol and excess NADPH (Figure S2). These were consistent with the differential gene expression results (Figure 1). In addition, flux through fatty acid elongation reactions catalyzed by desaturase enzymes such as stearyl CoA desaturase (SCD) was significantly modulated to enhance the ratio of saturated to monounsaturated fatty acids, and to generate excess NADPH (Figure 2E).

**FIGURE 3:**
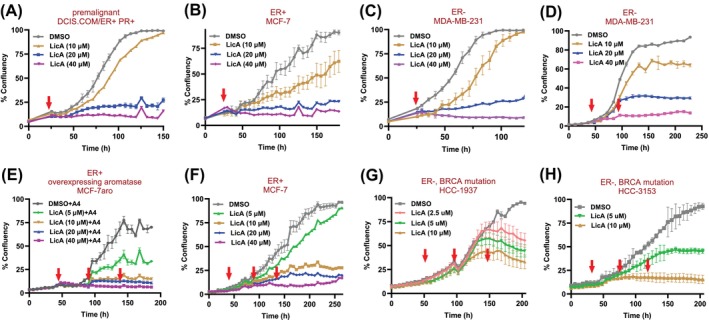
The effect of LicA on proliferation of ER+ and ER− breast cell lines, (A) premalignant DCIS.COM/ER+ PR+ treated with a single dose of various concentrations of LicA, (B) MCF‐7 treated with a single dose of various concentrations of LicA, (C) malignant MDA‐MB‐231 treated with a single dose of various concentrations of LicA, (D) MDA‐MB‐231 treated with repeated doses of various concentrations of LicA, (E) MCF‐7aro (MCF‐7 cells overexpressing aromatase) exposed to androstenedione (A4) to maintain their estrogen production and growth and treated with repeated doses of various concentrations of LicA, (F) MCF‐7 treated with repeated doses of various concentrations of LicA, (G) HCC‐1937 cells representing BRCA‐mutated triple negative breast cancer, treated with repeated doses of various concentrations of LicA, and (H) HCC‐3153 cells representing BRCA‐mutated triple negative breast cancer, treated with repeated doses of various concentrations of LicA. Repeated dosing of LicA exhibits sustained antiproliferative effects in aggressive cells. Red arrows show the time point(s) at which LicA or vehicle control was administered. Data represents mean ± SD of at least three independent replicates.

Licochalcone A suppresses the proliferation of pre‐malignant and malignant breast cells. Transcriptomic data and metabolic flux modeling indicated that LicA has antioxidant and anti‐inflammatory effects while reducing nucleotide production necessary for cell proliferation. We investigated the impact of LicA on breast cell proliferation at various concentrations (350 nM to 40 μM). A single dose of 20 μM or 40 μM effectively suppressed proliferation in DCIS.COM and DCIS.COM/ER+ PR+ cells for at least 4 days (Figure 3A). This observation held true for MCF‐7 and MDA‐MB‐231 cells (Figure 3B,C), which also responded to 10 μM of LicA, although the antiproliferative effect at this dose was not sustained following a single dose. Next, we tested repeated dosing of LicA, every 48 h for a maximum of 3 doses. Interestingly, two treatments of MDA‐MB‐231 cells with LicA (10 μM) 48 h apart until day 4 were able to sustainably retard proliferation until day 11 (Figure 3D) when we stopped our experiment. We also saw a reduction in the proliferation of MCF‐7 cells after 3 doses of LicA (10 μM) 48 h apart which was sustained at least until day 12, while MCF‐7aro cells, HCC1937, and HCC3153 cells were also very responsive to LicA (5 μM) (Figure 3E–H). Our BrdU assay confirmed an anti‐proliferative rather than a cytotoxic effect and the use of a ferroptosis inhibitor, ferrostatin, in combination with LicA showed that the antiproliferative effects are not due to ferroptosis (data not shown).

**FIGURE 4:**
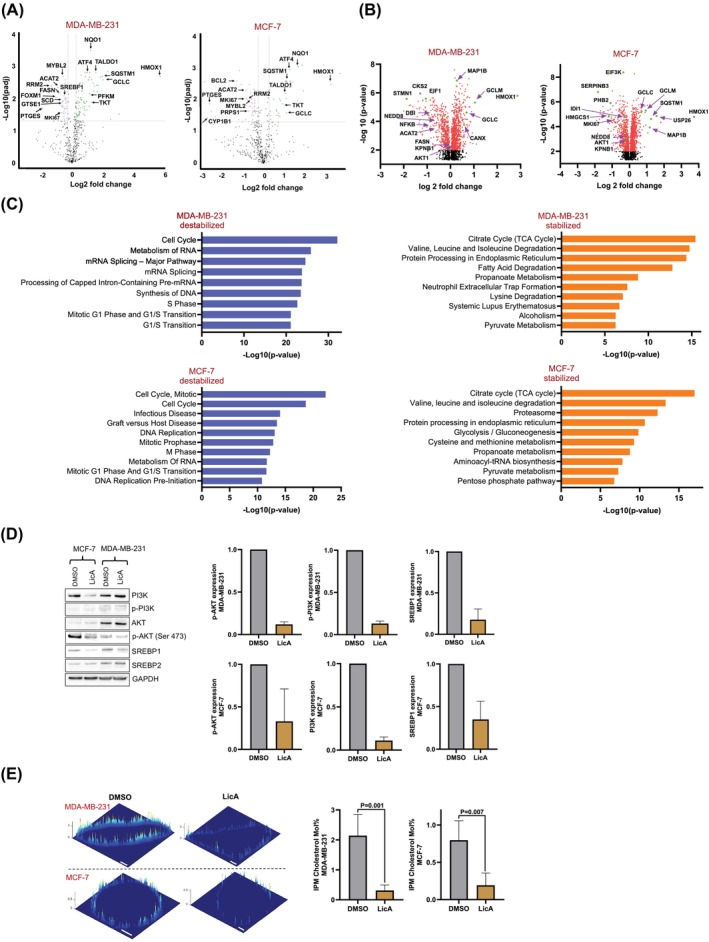
Changes in PI3K‐AKT‐SREBP1 lipogenesis in MCF‐7 (ER+) and MDA‐MB‐231 (ER−) treated with LicA (10 μM, 24 h). (A) NanoString metabolism pathway panel analysis shows the downregulation of lipogenesis, pro‐inflammatory, and pro‐proliferative genes and upregulation of antioxidant and anti‐inflammatory genes. Data represents the average of six analytical replicates of each cell line. (B) PISA proteomics volcano plots demonstrate the proteins significantly stabilized or destabilized after the labeled extracted peptides were analyzed using LC–MS/MS. Data represents the average of six analytical replicates of each cell line and was filtered for the PSM numbers >2 and the *p* <.05. (C) Pathway identification using Enrichr based on the significantly stabilized and destabilized proteins observed in PISA output. (D) Western blots: Quantification of the bands was performed by Image J. Data represent mean ± SD of three independent replicates. (E) Spatiotemporally resolved quantitative imaging of cholesterol in the inner leaflets of the plasma membranes (IPM). The IPM cholesterol concentrations before and after treatment with LicA (10 μM, 24 h) were calculated from the two‐channel cross‐sectional ratiometric images of representative cells at steady‐state. The *z*‐axis scale indicates the cholesterol concentration (mol%). A pseudo‐coloring scheme with red and blue representing the highest and the lowest concentration, respectively, is used to illustrate the spatial IPM cholesterol heterogeneity. Scale bars indicate 10 μm. A spatially averaged concentration (average ± SD from triplicate independent determinations with >10 cells per measurement) was calculated for each condition.

*Supporting evidence for antiproliferative effects* was provided with the NanoString data and the PISA proteomics results. Figure 4A demonstrates that the pro‐proliferative genes such as *BCL2* in MCF‐7 cells as well as *RRM2* and *MKI67* in *MCF‐7* and MDA‐MB‐231 cells were significantly downregulated. Consistent with our metabolism flux data in breast microstructures which showed reduced de novo nucleotide biosynthesis, we also observed significant downregulation of *PRPS1* in MCF‐7 cells (Figure 4A). Proteomics results (Figure 4B) further confirmed that in these cells, LicA destabilized enzymes essential for proliferation such as several EIFs and the proliferation marker MKI67, and that the majority of destabilized proteins were associated with cell cycle and proliferation (Figure 4C).

### 3.3 LicA downregulates SREBP1‐dependent lipogenesis and inflammation in MCF‐7 and MDA‐MB‐231 cell lines

The antiproliferative effects of LicA in BC cell lines (Figure 3) and the metabolic changes in breast tissue microstructures (Figure 1) prompted us to investigate the relationship between these effects and lipogenic metabolism and inflammation. Our NanoString metabolism panel results (Figure 4A) in MCF‐7 and MDA‐MB‐231 cells echoed our findings in high‐risk women's microstructures (Figure 1), showing downregulation of SREBP1‐dependent lipogenesis genes such as *ACAT2*, *FASN*, *SCD,* and the pro‐inflammatory *PTGES* (PGE2 synthase), alongside a marked upregulation of the anti‐inflammatory gene *HMOX1*.

*PISA proteomics results* aligned with our NanoString data, showing clear separation between LicA‐treated and DMSO‐treated cells in both ER+ and ER‐ cell lines (Figures S3A, B). Key proteins, including lipogenic enzymes (ACAT2, FASN, HMGCS1) and the inflammatory mediator NF‐kB1, were destabilized, along with significant suppression of karyopherin *β*1 (KPNB1), crucial for NF‐kB's nuclear translocation. LicA also destabilized NEDD8, which is important for SREBP1 stabilization. Conversely, the anti‐inflammatory protein HMOX1 was significantly stabilized (Figure 4B). It should be noted that some of the observed effects could be associated with the reduction of protein expression in conjunction with changes in protein stability.

*Analysis of proteomics results using Enrichr* revealed significant destabilization of cell cycle progression pathways (Figure 4C). Notably, while lipogenic metabolism was destabilized, the TCA cycle, branched amino acid degradation, and fatty acid degradation were the most stabilized pathways in both MDA‐MB‐231 and MCF‐7 cells (Figure 4C). Heatmaps of the differentially expressed proteins (Figure S3A, B) also align with these observations, demonstrating a major shift in the metabolic and inflammatory landscape. Correspondingly, western blotting (Figure 4D) showed that LicA treatment suppressed SREBP1 expression and phosphorylation of AKT at Ser437 and PI3K in both cell lines. Although PI3K levels decreased in MCF‐7 cells, there was a slight increase in MDA‐MB‐231 cells. The reduction in AKT phosphorylation occurred without changes in AKT expression. LicA suppresses local cholesterol levels in MCF‐7 and MDA‐MB‐231 cells. These changes align with the antiproliferative effects of LicA in these cell lines and are consistent with the previous reports of the inhibition of the PI3K‐AKT pathway to reduce the growth of MCF‐7 and MDA‐MB‐231 cells.^44^, ^45^

Transcriptomic data from the high‐risk women's breast microstructures and MCF‐7 and MDA‐MB‐231 cells treated with LicA, along with our proteomics observations showed significant suppression of SREBP1, SREBP2, and HMGCR with a shift in metabolism from lipogenesis to the TCA cycle, suggesting that cholesterol biosynthesis could be affected by LicA. Cellular free (unesterified) cholesterol is mainly found in the plasma membrane in mammalian cells.^46^ We thus performed spatiotemporally resolved in situ quantification of IPM cholesterol in MDA‐MB‐231 and MCF‐7 cells before and after LicA treatment.^41^ The results showed that the spatially averaged IPM cholesterol levels were significantly different between the two BC cell lines (Figure 4E). Importantly, when these cells were treated with LicA, their IPM cholesterol levels were drastically reduced, that is, 8‐fold in MDA‐MB‐231 cells (*n* = 14) and 4‐fold in MCF‐7 cells (*n* = 19) (Figure 4E). These reduced IPM cholesterol levels were comparable to those reported for unstimulated primary mammalian cells,^40^ demonstrating the efficacy of LicA in IPM cholesterol depletion.

### 3.4 Licochalcone A retards luminal and triple negative xenograft tumors in mice

**FIGURE 5:**
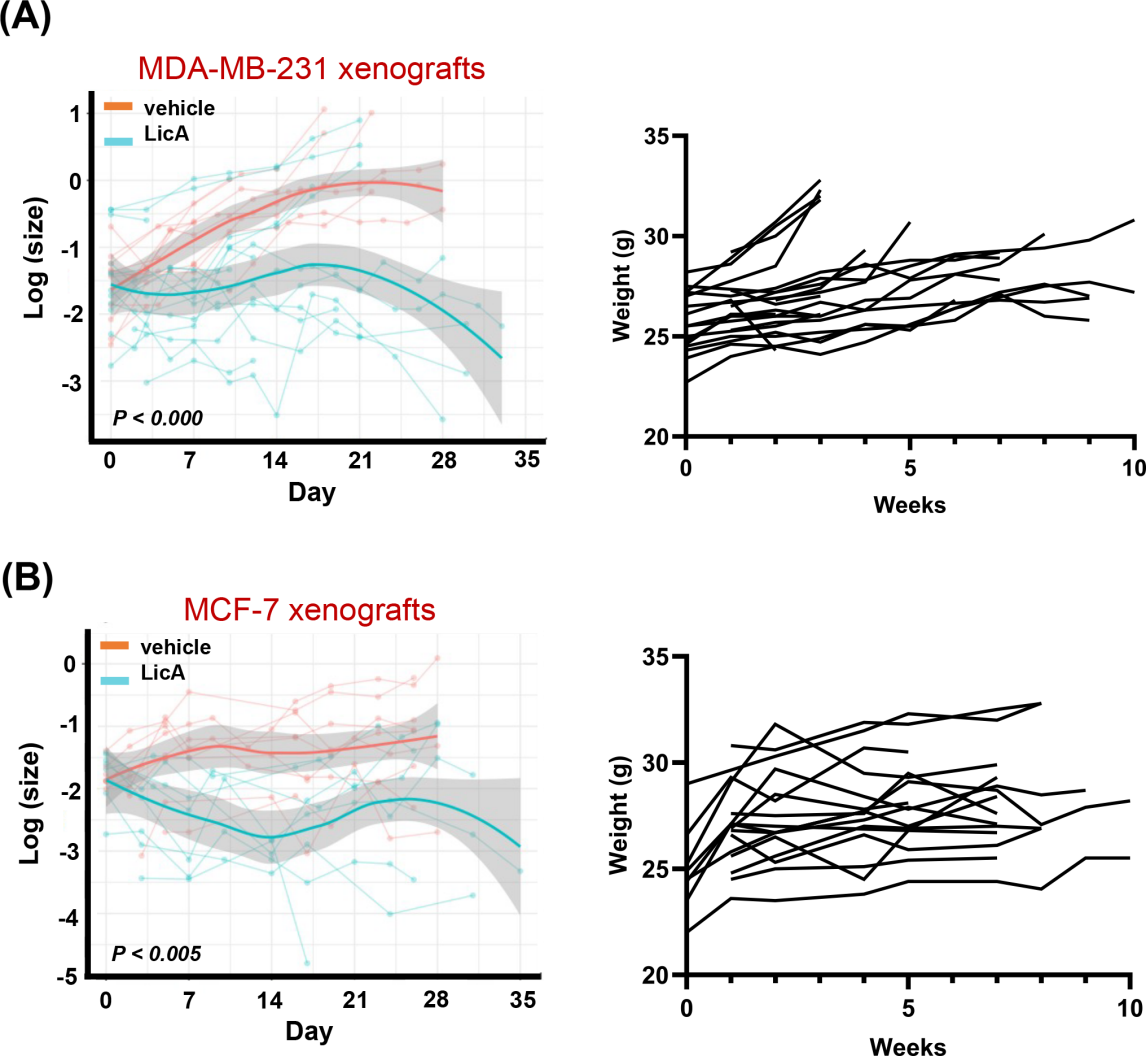
The effect of LicA on the rate of tumor growth in vivo. Xenografts of (A) MDA‐MB‐231 (*n* = 18 final tumor bearing animals) or (B) MCF‐7 cells (*n* = 12 final tumor bearing animals) were performed in the right (Rt) and left (Lt) flanks of ovary‐intact athymic nude mice. After the tumors reached 0.8 cm in diameter, subcutaneous administration of LicA (80 mg/kg.day) commenced and continued for 28 days. A significant reduction in the rate of tumor growth was observed in the LicA treated versus vehicle treated animals.

Given the antiproliferative effects of LicA in breast cell lines (Figure 3) and the downregulation of proliferation‐related genes (Figure 4), we assessed its effects in xenograft models of ER+ and ER− mammary cancers. In both xenograft models, analysis of results included all the animals assigned to each experimental group. In animals bearing MDA‐MB‐231 (ER−) xenografts, daily treatment with LicA (80 mg/kg. day, s.c.) for 28 days significantly (*p* <.000) reduced the growth rate of tumors in 7/9 (78%) of animals (Figure 5A). In the animals bearing MCF‐7 xenograft tumors, treatment with LicA led to a significant (*p* <.005) reduction in the rate of tumor growth in all 6/6 (100%) animals, with a relatively similar responsiveness to treatment (Figure 5B).

## 4 DISCUSSION

One of the hallmarks of cancer and an essential step in malignant transformation is metabolic reprogramming that promotes malignant cell survival.^19^ A main mechanism for this effect is the reprogramming of lipid metabolic pathways. Within this major pathway, the *SREBF1* gene encodes SREBP1a and SREBP1c proteins, transcription factors that regulate lipogenesis. *SREBF1* expression is significantly higher in breast tumors compared to normal tissue adjacent to the tumor and in healthy breast tissues of 924 patients in the GTEx and TCGA datasets.^47^ Conversely, the downregulation of SREBP1 not only decreases lipogenesis but is also associated with bone protection.^48^, ^49^ It is known that SREBP1 regulates the expression of lipogenic enzymes, FASN, ACAT2, ACC1, and ACLY which are essential for the survival of BC cells.^13^, ^50^ There is also a strong association between breast tissue stiffness (a risk factor for breast carcinogenesis) and SREBP1 activity.^14^, ^51^ Recent studies have demonstrated that activation of NF‐kB‐dependent inflammation happens in part through the translocation of the inactive SREBP1‐NF‐kB complex to the Golgi apparatus and the subsequent activation of SREBP1 mediated by the SREBP cleavage activating protein (Scap)^15^ suggesting a close connection between SREBP1‐dependent metabolism and NF‐kB‐dependent inflammation. An inflamed environment supports the growth of malignant cells. Upstream of SREBP1, EGFR activation and PI3K‐AKT–mTOR mutation, as well as mTORC1 activity lead to enhanced SREBP1 expression and consequent increase in SCD activity known to be important for cancer initiation, promotion, and invasion.^52^, ^53^

**FIGURE 6:**
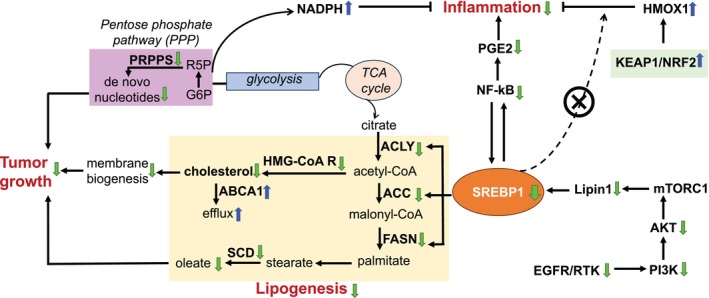
Suppressing lipogenesis and inflammation, mediated by the central role of SREBP1 regulation by LicA, leads to reduced proliferation in the breast. Colored arrows represent the effects of LicA.

We used breast microstructures from the opposite breasts of women with breast cancer, since these represent normal/benign tissue that is known to be at increased risk of a second new breast cancer. LicA significantly lowered *SREBF1*, as well as the expression of several of its upstream effectors such as *EGFR*, *PI3K*, *AKT*, and *mTORC1*, in addition to *SCD* which is downstream of *SREBF1*. These observations extended to MDA‐MB‐231 and MCF‐7 cells, where transcriptomics and proteomics data clearly demonstrate significant reduction of lipogenesis signals regulated by SREBP1, in concert with a significant anti‐inflammatory response with suppression of PGE2 biosynthesis and a profound increase in HMOX1 expression. Interestingly, SREBP1 has response elements at the promoter region of *HMOX1*, that is, downregulation of SREBP1 is expected to lower *HMOX1* expression; however, in our results, HMOX1 at gene and protein levels is profoundly enhanced. This could be associated with the effect of LicA on the KEAP1‐NRF2 pathway that we had reported^20^ which activates HMOX1 and compensates for its likely reduction due to SREBP1 downregulation (Figure 6). In our results, the significant decrease in the expression of SREBP1 is accompanied by reduced stabilization of NEDD8 protein which is important for the stabilization of SREBP1 through posttranslational neddylation, a process linked to BC aggressiveness.^54^ In addition, lowered SREBP1 expression along with the reduced phosphorylation of its upstream effectors PI3K and AKT and the profound drop in cellular cholesterol levels align with the metabolic shift observed in our proteomics studies. The combined effects of LicA on shifting metabolism away from lipogenesis along with significant destabilization of the cell cycle further validate that LicA reprograms cellular metabolism in favor of reducing cell proliferation. These results demonstrate that the axis of PI3K‐AKT‐SREBP1‐NF‐kB is the mechanism by which LicA exerts its antiproliferative and tumor‐suppressive effects. LicA's precise target in this axis is yet to be elucidated.

Another important gene in lipid metabolism, *SREBF2* encoding the SREBP2 protein, is under the control of SREBP1 and primarily regulates cholesterol biosynthesis and homeostasis.^55^ It is known that enhanced expression of *SREBF2* promotes the progression of several types of cancers.^55^ *SREBF2* is significantly higher in breast tumors compared to healthy tissues of 924 patients in GTEx and TCGA samples.^47^ *SREBF2* regulates key genes in the mevalonate pathway such as *MVD*, *MVK*, *SQLN*, and *HMGCR*, involved in cholesterol biosynthesis. It was recently reported that cholesterol [Chol]_i_ in the inner leaflet of the plasma membrane (IPM) of mammalian cells is directly and quantitatively related to the proliferative activity of cells.^41^ The significant reduction in cholesterol biosynthesis in the inner leaflet of the plasma membrane which we depicted by measuring [Chol]_i_ in MCF‐7 and MDA‐MB‐231 cells further validated the metabolic reprogramming. The difference we found in the [Chol]_i_ between MCF‐7 and MDA‐MB‐231 cells reflects differential reprogramming of cholesterol homeostasis in these two cell lines.

Interestingly, changes in the SREBP2 expression in women's breast microstructures and in malignant breast cell lines MCF‐7 and MDA‐MB‐231 differ. In women's breast microstructures which are nonmalignant and composed of a mixture of various cell types of breast tissue LicA profoundly downregulated SREBP2; however, in malignant breast cell lines there was no significant change in *SREBF2* or SREBP2 levels, despite the suppression of IPM cholesterol. Thus, LicA reduces lipogenesis in non‐malignant high‐risk breast through reducing the expression of both SREBP1 and SREBP2 while its effects on BC cells are primarily mediated by SREBP1. A recent study showed that concerted SREBP1–NF‐kB modulation might exert responses independent of SREBP2 levels or activity.^15^ In other words, it is highly likely that the reduced proliferation in cancer cells is primarily regulated by the SREBP1–NF‐kB complex, through direct interaction with Scap.^15^ However, in non‐malignant high‐risk breast tissue the additional reduction in SREBP2 levels, reinforces the prevention of cellular transformation. It is also likely that despite the tumor suppressive effects of LicA in both MCF‐7 and MDA‐MB‐231 xenografts, the precise LicA target(s) are cell type‐dependent, since there were differences in SREBP1, PI3K, and AKT protein level changes in these cell lines.

We found that metabolic flux in high‐risk women's breast exposed to LicA was not only in favor of antioxidant and anti‐inflammatory pathways; flux was also reprogrammed to minimize cell proliferation, the effect that we confirmed in a series of orthogonal experiments.

In addition to the SREBP1‐dependent effects that lead to reduced cell proliferation, our data once again show LicA limits oxidative and inflammatory stress through the activity of NRF2 and suppressing NF‐kB pathways in the breast tissue microstructures of at‐risk women providing cytoprotective effects. Moreover, the significant expression of SQSTM1 in the breast microstructures, as well as in cell lines treated with LicA supports its previously reported bone protective effects.^25^, ^27^

Overall, our data suggest that LicA‐based interventions will effectively reduce BC risk (Figure 6). LicA protects against oxidative and inflammatory stress, deprives cancer stem cells of their favored inflammatory environment, and lowers lipogenesis and proliferation. LicA complies with Lipinski's rule of 5 (RO5) in drug discovery which defines LicA as orally active. Based on this notion and its encouraging biological activity, LicA is a good candidate for further development as a novel BC risk reduction drug with sufficient efficacy, reduced toxicity, and therefore, is anticipated to have greater acceptance by women at increased risk. Such an agent with effects against both ER+ and ER‐ subtypes and demonstrated bone protective properties is predicted to have better success and a larger impact than the current agents. We are now planning the next series of studies including evaluations in immunocompetent models of cancer prevention, and rigorous formulation and pharmacokinetic evaluations for oral delivery, that are required for translation to clinical testing.

## AUTHOR CONTRIBUTIONS

**Atieh Hajirahimkhan:** Conceptualization; investigation; funding acquisition; writing – original draft; methodology; validation; visualization; writing – review and editing; formal analysis; project administration; data curation; resources. **Elizabeth T. Bartom:** Formal analysis; data curation; investigation; validation; methodology. **Carolina H. Chung:** Formal analysis; data curation; investigation; visualization. **Xingyu Guo:** Investigation; formal analysis; visualization. **Kyli Berkley:** Investigation; visualization; formal analysis. **Seyyedmohsen Hosseinibarkooie:** Formal analysis; writing – review and editing. **Zahra Assadi:** Formal analysis. **Shao Huan Samuel Weng:** Writing – review and editing; formal analysis; data curation. **Raymond Moellering:** Supervision. **Oukseub Lee:** Methodology. **Ruohui Chen:** Formal analysis; data curation; visualization. **Wonhwa Cho:** Supervision; methodology; writing – review and editing; resources. **Sriram Chandrasekaran:** Methodology; writing – review and editing; supervision; resources. **Susan E. Clare:** Writing – review and editing; supervision; resources. **Seema A. Khan:** Writing – review and editing; supervision; resources.

## FUNDING INFORMATION

American Cancer Society postdoctoral fellowship 131667‐PF‐18‐049‐01NEC to AH, Robert H Lurie Comprehensive Cancer Center Translational Bridge Fellowship to AH, National Cancer Institute T32 postdoctoral fellowship to AH, Northwestern University Department of Surgery Seed funding to AH, Bramsen‐Hamill Foundation funding to SAK, The National Institutes of Health grant R35GM122530 to WC.

## CONFLICT OF INTEREST STATEMENT

The authors declare that they have nothing to disclose and no competing interests.

## ETHICS STATEMENT

Institutional Review Board (IRB) approval (IRB STU00202331) was obtained from Northwestern University prior to obtaining informed consents from patients and collecting samples. All experiments were conducted in accordance with the approved protocol and guidelines. All the in vivo studies and procedures were performed under the approved Northwestern University IACUC protocol # IS00013602.

## Supporting information


**Table S1:** Quality statistics of transcriptomic studies presented in Figures 1 and 2.

**Table S2:** NanoString n‐Counter pathway panel genes, probes, and annotations associated with data in Figure 1.

**Table S3:** Raw gene expression reads associated with the data presented in Figures 1 and 2.

**Data S1.** Supporting Information.

## Data Availability

The RNA Sequencing data generated in this study is available in GEO under accession number GSE290030. The mass spectrometry data generated in this study has been deposited in the PRIDE database with dataset identifier PXD061086. Other (deidentified) data that support the findings of this study are available from the corresponding author upon request.

## References

[1] Khan SA . Breast cancer risk reduction: current status and emerging trends to increase efficacy and reduce toxicity of preventive medication. Surg Oncol Clin N Am. 2023;32:631‐646. 37714633 10.1016/j.soc.2023.05.001

[2] Britt KL , Cuzick J , Phillips KA . Key steps for effective breast cancer prevention. Nat Rev Cancer. 2020;20:417‐436. 32528185 10.1038/s41568-020-0266-x

[3] Jahan N , Jones C , Rahman RL . Endocrine prevention of breast cancer. Mol Cell Endocrinol. 2021;530:111284. 33882282 10.1016/j.mce.2021.111284

[4] Sakr RA , Weigelt B , Chandarlapaty S , et al. PI3K pathway activation in high‐grade ductal carcinoma in situ—implications for progression to invasive breast carcinoma. Clin Cancer Res. 2014;20:2326‐2337. 24634376 10.1158/1078-0432.CCR-13-2267 PMC4015460

[5] Bouamar H , Broome LE , Lathrop KI , et al. mTOR inhibition abrogates human mammary stem cells and early breast cancer progression markers. Breast Cancer Res. 2023;25:131. 37904250 10.1186/s13058-023-01727-z PMC10614399

[6] Mazumdar A , Tahaney WM , Hill JL , et al. Targeting the mTOR pathway for the prevention of ER‐negative breast cancer. Cancer Prev Res (Phila). 2022;15:791‐802. 35981902 10.1158/1940-6207.CAPR-22-0106 PMC9762336

[7] Ku AT , Young AIJ , Ibrahim AA , et al. Short‐term PI3K inhibition prevents breast cancer in preclinical models. Cancer Prev Res. 2023;16:65‐73. 10.1158/1940-6207.CAPR-22-0275 PMC9905287 36343340

[8] Chen G , Ding XF , Pressley K , et al. Everolimus inhibits the progression of ductal carcinoma in situ to invasive breast cancer via downregulation of MMP9 expression. Clin Cancer Res. 2020;26:1486‐1496. 31871301 10.1158/1078-0432.CCR-19-2478 PMC8800315

[9] Nye L , Khan SA . Everolimus for estrogen receptor‐negative breast cancer prevention: a journey begun? Cancer Prev Res. 2022;15:787‐789. 10.1158/1940-6207.CAPR-22-0419 36453053

[10] Goodwin PJ , Chen BE , Gelmon KA , et al. Effect of metformin versus placebo on new primary cancers in Canadian cancer trials group MA.32: a secondary analysis of a phase III randomized double‐blind trial in early breast cancer. J Clin Oncol. 2023;41:5356‐5362. 37695982 10.1200/JCO.23.00296 PMC10713140

[11] Hershman DL , Chen BE , Sathe C , et al. Metformin, placebo, and endocrine therapy discontinuation among participants in a randomized double‐blind trial of metformin vs placebo in hormone receptor‐positive early‐stage breast cancer (CCTG MA32). Breast Cancer Res Treat. 2023;200:93‐102. 37157006 10.1007/s10549-023-06922-2

[12] Zipinotti Dos Santos D , de Souza JC , Pimenta TM , et al. The impact of lipid metabolism on breast cancer: a review about its role in tumorigenesis and immune escape. Cell Commun Signal. 2023;21:161. 37370164 10.1186/s12964-023-01178-1 PMC10304265

[13] Bao J , Zhu L , Zhu Q , Su J , Liu M , Huang W . SREBP‐1 is an independent prognostic marker and promotes invasion and migration in breast cancer. Oncol Lett. 2016;12:2409‐2416. 27703522 10.3892/ol.2016.4988 PMC5038874

[14] Bertolio R , Napoletano F , Mano M , et al. Sterol regulatory element binding protein 1 couples mechanical cues and lipid metabolism. Nat Commun. 2019;10:1326. 30902980 10.1038/s41467-019-09152-7 PMC6430766

[15] Fei X , Huang J , Li F , et al. The Scap‐SREBP1‐S1P/S2P lipogenesis signal orchestrates the homeostasis and spatiotemporal activation of NF‐kappaB. Cell Rep. 2023;42:112586. 37267109 10.1016/j.celrep.2023.112586

[16] Jeon YG , Kim YY , Lee G , Kim JB . Physiological and pathological roles of lipogenesis. Nat Metab. 2023;5:735‐759. 37142787 10.1038/s42255-023-00786-y

[17] Lee CM , Fang S . Fat biology in triple‐negative breast cancer: immune regulation, fibrosis, and senescence. J Obes Metab Syndr. 2023;32:312‐321. 38014425 10.7570/jomes23044 PMC10786212

[18] Chang MC , Eslami Z , Ennis M , Goodwin PJ . Crown‐like structures in breast adipose tissue of breast cancer patients: associations with CD68 expression, obesity, metabolic factors and prognosis. NPJ Breast Cancer. 2021;7:97. 34294716 10.1038/s41523-021-00304-x PMC8298396

[19] Hanahan D . Hallmarks of cancer: new dimensions. Cancer Discov. 2022;12:31‐46. 35022204 10.1158/2159-8290.CD-21-1059

[20] Hajirahimkhan A , Simmler C , Dong H , et al. Induction of NAD(P)H:quinone oxidoreductase 1 (NQO1) by *glycyrrhiza* species used for Women's health: differential effects of the Michael acceptors Isoliquiritigenin and Licochalcone a. Chem Res Toxicol. 2015;28:2130‐2141. 26473469 10.1021/acs.chemrestox.5b00310 PMC4898475

[21] Boonchai W , Varothai S , Winayanuwattikun W , Phaitoonvatanakij S , Chaweekulrat P , Kasemsarn P . Randomized investigator‐blinded comparative study of moisturizer containing 4‐t‐butylcyclohexanol and licochalcone a versus 0.02% triamcinolone acetonide cream in facial dermatitis. J Cosmet Dermatol. 2018;17:1130‐1135. 29411520 10.1111/jocd.12499

[22] Jia T , Qiao J , Guan D , Chen T . Anti‐inflammatory effects of Licochalcone a on IL‐1beta‐stimulated human osteoarthritis chondrocytes. Inflammation. 2017;40:1894‐1902. 28756519 10.1007/s10753-017-0630-5

[23] Hajirahimkhan A , Howell C , Bartom ET , et al. Breast cancer prevention with liquiritigenin from licorice through the inhibition of aromatase and protein biosynthesis in high‐risk women's breast tissue. Sci Rep. 2023;13:8734. 37253812 10.1038/s41598-023-34762-z PMC10229614

[24] Wang S , Dunlap TL , Huang L , et al. Evidence for Chemopreventive and resilience activity of licorice: glycyrrhiza glabra and G. Inflata extracts modulate estrogen metabolism in ACI rats. Cancer Prev Res (Phila). 2018;11:819‐830. 30287522 10.1158/1940-6207.CAPR-18-0178 PMC6435032

[25] Yan Z , Qi W , Zhan J , et al. Activating Nrf2 signalling alleviates osteoarthritis development by inhibiting inflammasome activation. J Cell Mol Med. 2020;24:13046‐13057. 32965793 10.1111/jcmm.15905 PMC7701566

[26] Guo W , Liu B , Yin Y , et al. Licochalcone a protects the blood‐milk barrier integrity and relieves the inflammatory response in LPS‐induced mastitis. Front Immunol. 2019;10:287. 30858849 10.3389/fimmu.2019.00287 PMC6398509

[27] Ming L , Jin F , Huang P , et al. Licochalcone a up‐regulates of FasL in mesenchymal stem cells to strengthen bone formation and increase bone mass. Sci Rep. 2014;4:7209. 25428397 10.1038/srep07209 PMC4245531

[28] Cuadrado A , Rojo AI , Wells G , et al. Therapeutic targeting of the NRF2 and KEAP1 partnership in chronic diseases. Nat Rev Drug Discov. 2019;18:295‐317. 30610225 10.1038/s41573-018-0008-x

[29] Kim SH , Yang M , Xu JG , Yu X , Qian XJ . Role of licochalcone a on thymic stromal lymphopoietin expression: implications for asthma. Exp Biol Med. 2015;240:26‐33. 10.1177/1535370214545020 PMC4935181 25055998

[30] Funakoshi‐Tago M , Nakamura K , Tsuruya R , et al. The fixed structure of Licochalcone a by alpha, beta‐unsaturated ketone is necessary for anti‐inflammatory activity through the inhibition of NF‐kappaB activation. Int Immunopharmacol. 2010;10:562‐571. 20153843 10.1016/j.intimp.2010.02.003

[31] Furusawa J , Funakoshi‐Tago M , Tago K , et al. Licochalcone a significantly suppresses LPS signaling pathway through the inhibition of NF‐kappaB p65 phosphorylation at serine 276. Cell Signal. 2009;21:778‐785. 19168128 10.1016/j.cellsig.2009.01.021

[32] Villanueva H , Grimm S , Dhamne S , et al. The emerging roles of steroid hormone receptors in ductal carcinoma in situ (DCIS) of the breast. J Mammary Gland Biol Neoplasia. 2018;23:237‐248. 30338425 10.1007/s10911-018-9416-0 PMC6244884

[33] Lui K , Tamura T , Mori T , Zhou D , Chen S . MCF‐7aro/ERE, a novel cell line for rapid screening of aromatase inhibitors, ERalpha ligands and ERRalpha ligands. Biochem Pharmacol. 2008;76:208‐215. 18550029 10.1016/j.bcp.2008.04.011 PMC2587126

[34] Tanos T , Sflomos G , Echeverria PC , et al. Progesterone/RANKL is a major regulatory axis in the human breast. Sci Transl Med. 2013;5:182ra55. 10.1126/scitranslmed.3005654 23616122

[35] Dobin A , Davis CA , Schlesinger F , et al. STAR: ultrafast universal RNA‐seq aligner. Bioinformatics. 2013;29:15‐21. 23104886 10.1093/bioinformatics/bts635 PMC3530905

[36] Robinson MD , McCarthy DJ , Smyth GK . edgeR: a Bioconductor package for differential expression analysis of digital gene expression data. Bioinformatics. 2010;26:139‐140. 19910308 10.1093/bioinformatics/btp616 PMC2796818

[37] Chandrasekaran S . A protocol for the construction and curation of genome‐scale integrated metabolic and regulatory network models. Methods Mol Biol. 2019;1927:203‐214. 30788794 10.1007/978-1-4939-9142-6_14

[38] Shen F , Cheek C , Chandrasekaran S . Dynamic network modeling of stem cell metabolism. Methods Mol Biol. 2019;1975:305‐320. 31062316 10.1007/978-1-4939-9224-9_14

[39] Mirzaei M , Pascovici D , Wu JX , et al. TMT one‐stop shop: from reliable sample preparation to computational analysis platform. Methods Mol Biol. 2017;1549:45‐66. 27975283 10.1007/978-1-4939-6740-7_5

[40] Buwaneka P , Ralko A , Liu SL , Cho W . Evaluation of the available cholesterol concentration in the inner leaflet of the plasma membrane of mammalian cells. J Lipid Res. 2021;62:100084. 33964305 10.1016/j.jlr.2021.100084 PMC8178126

[41] Liu SL , Sheng R , Jung JH , et al. Orthogonal lipid sensors identify transbilayer asymmetry of plasma membrane cholesterol. Nat Chem Biol. 2017;13:268‐274. 28024150 10.1038/nchembio.2268 PMC5912897

[42] Chung CH , Lin DW , Eames A , Chandrasekaran S . Next‐generation genome‐scale metabolic modeling through integration of regulatory mechanisms. Metabolites. 2021;11:11. 10.3390/metabo11090606 PMC8470976 34564422

[43] King ZA , Lu J , Drager A , et al. BiGG models: a platform for integrating, standardizing and sharing genome‐scale models. Nucleic Acids Res. 2016;44:D515‐D522. 26476456 10.1093/nar/gkv1049 PMC4702785

[44] Khan MA , Jain VK , Rizwanullah M , Ahmad J , Jain K . PI3K/AKT/mTOR pathway inhibitors in triple‐negative breast cancer: a review on drug discovery and future challenges. Drug Discov Today. 2019;24:2181‐2191. 31520748 10.1016/j.drudis.2019.09.001

[45] Deng S , Wang L , Tian S , et al. Thiazolidinedione‐based structure modification of ergosterol peroxide provides thiazolidinedione‐conjugated derivatives as potent agents against breast cancer cells through a PI3K/AKT/mTOR pathway. Bioorg Med Chem. 2025;117:118007. 39577295 10.1016/j.bmc.2024.118007

[46] van Meer G . Dynamic transbilayer lipid asymmetry. Cold Spring Harb Perspect Biol. 2011;3:a004671. 21436058 10.1101/cshperspect.a004671 PMC3101844

[47] Jezequel P , Gouraud W , Ben Azzouz F , et al. Bc‐GenExMiner 4.5: new mining module computes breast cancer differential gene expression analyses. Database. 2021;2021:baab007. 33599248 10.1093/database/baab007 PMC7904047

[48] Zheng ZG , Zhang X , Zhou YP , et al. Anhydroicaritin, a SREBPs inhibitor, inhibits RANKL‐induced osteoclastic differentiation and improves diabetic osteoporosis in STZ‐induced mice. Eur J Pharmacol. 2017;809:156‐162. 28501578 10.1016/j.ejphar.2017.05.017

[49] Inoue K , Imai Y . Fatostatin, an SREBP inhibitor, prevented RANKL‐induced bone loss by suppression of osteoclast differentiation. Biochim Biophys Acta. 2015;1852:2432‐2441. 26319416 10.1016/j.bbadis.2015.08.018

[50] Menendez JA , Lupu R . Fatty acid synthase and the lipogenic phenotype in cancer pathogenesis. Nat Rev Cancer. 2007;7:763‐777. 17882277 10.1038/nrc2222

[51] Perone Y , Farrugia AJ , Rodriguez‐Meira A , et al. SREBP1 drives Keratin‐80‐dependent cytoskeletal changes and invasive behavior in endocrine‐resistant ERalpha breast cancer. Nat Commun. 2019;10:2115. 31073170 10.1038/s41467-019-09676-y PMC6509342

[52] Porstmann T , Griffiths B , Chung YL , et al. PKB/Akt induces transcription of enzymes involved in cholesterol and fatty acid biosynthesis via activation of SREBP. Oncogene. 2005;24:6465‐6481. 16007182 10.1038/sj.onc.1208802

[53] Ascenzi F , De Vitis C , Maugeri‐Sacca M , Napoli C , Ciliberto G , Mancini R . SCD1, autophagy and cancer: implications for therapy. J Exp Clin Cancer Res. 2021;40:265. 34429143 10.1186/s13046-021-02067-6 PMC8383407

[54] Heo MJ , Kang SH , Kim YS , et al. UBC12‐mediated SREBP‐1 neddylation worsens metastatic tumor prognosis. Int J Cancer. 2020;147:2550‐2563. 32449166 10.1002/ijc.33113

[55] Xue L , Qi H , Zhang H , et al. Targeting SREBP‐2‐regulated mevalonate metabolism for cancer therapy. Front Oncol. 2020;10:1510. 32974183 10.3389/fonc.2020.01510 PMC7472741

