# Supplementary material for: Reprogramming SREBP1‐dependent lipogenesis and inflammation in high‐risk breast with licochalcone A: A novel path to cancer prevention
Source: Int J Cancer. 2025 Nov 27;158(7):1927–40. doi: 10.1002/ijc.70226 (PMC12875168; doi:10.1002/ijc.70226)

Supplementary Materials

Reprogramming SREBP1-dependent lipogenesis and inflammation in high-risk breast with licochalcone A: a novel path to cancer prevention

Atieh Hajirahimkhan, Elizabeth T. Bartom, Carolina H Chung, Xingyu Guo, Kyli Berkley, Seyyedmohsen Hosseinibarkooie, Zahra Assadi, Shao Huan Samuel Weng, Raymond Moellering, Oukseub Lee, Ruohui Chen, Wonhwa Cho, Sriram Chandrasekaran, Susan E. Clare, Seema A. Khan.

Table of Contents:

Supplementary Materials and Methods

Supplementary Figure S1A

Supplementary Figure S1B

Supplementary Figure S2

Supplementary Figure S3A

Supplementary Figure S3B

Supplementary Table S1 (provided as a separate file)

Supplementary Table S2 (provided as a separate file)

Supplementary Table S3 (provided as a separate file)

Supplementary Materials and Methods.

Chemicals and Materials. All chemicals and reagents were purchased from Sigma-Aldrich (St. Louis, MO), unless otherwise indicated. MammoCult media kit, heparin, and hydrocortisone were purchased from Stem Cell Technologies (Vancouver, BC. Canada). F12-K nutrient mix (Kaighn's) medium was acquired from Gibco (Dublin, Ireland). Fetal bovine serum (FBS) was purchased from Atlanta Biologicals (Norcross, GA). Collagenase I was purchased from Sigma-Aldrich (St. Louis, MO). Licochalcone A (LicA) was acquired from Med Chem Express (Monmouth Junction, NJ). Direct-Zol RNA prep kit was acquired from Zymoresearch (Irvine, CA). TRIzol was obtained from Invitrogen (Waltham, MA). RNAeasy mini prep kit was purchased from QIAGEN (Germantown, MD). RNA concentration and clean up kit was acquired from Norgen Biotek (ON, Canada). KAPA RNA HyperPrep library preparation kit was obtained from Roche (Madison, WI). Unique dual adaptors for next generation sequencing (NGS) and PCR reagents, primers, and master mix were purchased from Integrated DNA Technologies (Coralville, IA). Antibodies were obtained from Abcam (Cambridge, UK). Western blot reagents, buffers, supplies were purchased from Thermo Fisher Scientific (Hanover Park, IL). S-Trap column was purchased from ProtiFi (Fairport, NY). Mass spec grade Trypsin/Lys-C mix was obtained from Promega (Madison, WI).

Proliferation assay. We seeded pre-malignant DCIS.COM and DCIS.COM/ER+ PR+ cell lines as well as malignant MCF-7 (ER+ PR+), MDA-MB-231 (ER- PR-), MCF-7aro (ER+ PR+), HCC-1937 (ER- PR-, BRCA1 mutated), and HCC-3153 (ER- PR-, BRCA mutated1) cells with the density of 2.5 x 10^3 cells/well in their appropriate media in 96-well plates. We placed the plate in IncuCyte instrument for continued live cell imaging every 6 h. When cells reached 30% confluency, eight different concentrations of LicA ranging from 350 nM to 40 μM were added to the designated wells. We added comparable dilutions of DMSO to the wells allocated for vehicle control. In the case of MCF-7aro cells which overexpress *cyp19a1* (aromatase), the dosing of LicA or DMSO was performed in the presence of androstenedione, the substrate for aromatase. We continued live cell imaging for a minimum of 4 days post treatment. When we found out that a single low dose is not sufficient for sustained antiproliferation, we added the treatment and control every 48 h for 6 days and continued live cell imaging for a maximum of 12 days. Data was analyzed using Zoom software to quantify the images and were plotted as mean ± SEM of at least two independent measurements.

Proteome Integral Solubility Alterations (PISA) proteomics. MCF-7 and MDA-MB-231 cells were treated with LicA (10 μM) for 24 h. Cells were detached with non-enzymatic dissociation buffer and centrifuged at 300 x g for 4 min. The cell pellets were reconstituted in PBS containing protease and phosphatase inhibitors. This step was repeated. The final pellet was resuspended in PBS with inhibitors and was distributed into PCR tubes. Thermal denaturation was performed with a steady temperature at 40, 42, 45, 50, 55, 60, 63, and 65°C for 3 mins in parallel in a thermal cycler. Immediately after heating, the samples were removed and incubated at room temperature for 3 min before they were snap-frozen with liquid nitrogen. Two cycles of freeze-thaw using liquid nitrogen and a heating block set at 25°C were conducted to ensure a uniform temperature between tubes. After the vortex, the lysates were centrifuged at 20,000 x g for 20 min at 4°C to pellet the debris with precipitated and aggregated proteins. While on ice, the supernatant with the soluble protein fraction was carefully removed to a new tube. BCA assay quantified the protein concentration in the supernatant for the two lowest temperatures. The average of the two lowest temperatures was calculated and the volume equivalent to 30 μg of protein in the lowest temperature was moved from each temperature fraction into a clean tube. Following the distribution of protein, each tube was brought to a final volume of 100 μL by the addition of S-Trap lysis buffer (5% SDS, 50mM TEAB, pH 7.55, adjust pH with 12% phosphoric acid) with inhibitors. Samples were reduced with 1M Tris(2-carboxyethyl) phosphine

hydrochloride (TCEP; 20 mM final) and incubated at 65°C for 30 mins. Alkylation was then performed with 0.5M iodoacetamide (IAA; 80 mM final) at room temperature in the dark for 30 mins. Samples were acidified with 12% phosphoric acid to a final concentration of 1.2% and diluted sixfold with S-Trap binding buffer (methanol containing 100 mM TEAB, pH adjusted to 7.2 using 12% phosphoric acid). Samples were then loaded onto an S-Trap micro column in 100 µL increments using a microcentrifuge with the flow-through collected as waste.

SDS was completely removed by washing the filter four times with 160 µL of S-Trap binding buffer. The purified protein on the filter was then digested with 2 µg of trypsin/Lys-C in 50 mM ammonium bicarbonate and 0.5 mM $CaCl_2$ at 37°C overnight. Digested peptides were collected in three sequential washes: 40 µL of 50 mM ammonium bicarbonate, followed by 40 µL of 0.15% formic acid, and finally 35 µL of 0.15% formic acid in 50% acetonitrile. The eluate was dried under vacuum and stored at −80°C until analysis.

The dried material was reconstituted in 100 mM TEAB with pH 8.5. TMT reagent was added to the peptide solution and incubated for 1 h at room temperature. The reaction was quenched with 5% hydroxylamine. Peptides were cleaned using a C18 desalting column. The labeled peptides were fractionated using high pH reverse-phase chromatography to reduce sample complexity. The fractionated peptides were analyzed using LC-MS/MS, and MS3 was used for quantification and identification of peptides. Proteome Discoverer was used for search[45]. Statistical analysis was performed using Python. The differentially expressed proteins were also analyzed by Enrichr to obtain significantly stabilized and destabilized pathways.

Western Blot. The Western blot analysis was performed as described before.[25] After 48 h treatment of each compound, the cells were lysed with RIPA buffer (50 mM Tris-HCl pH 7.5, 0.1% SDS, 1% Triton X-100, 150 mM NaCl, 0.5% Sodium deoxycholate, and 2 mM EDTA) containing protease/phosphatase inhibitor cocktail (Thermo Fisher Scientific, Waltham, MA, USA) at 4°C for 1 h. The supernatants were collected and quantified using BCA protein assay kit (Thermo Fisher Scientific) according to the manufacturer's instructions. To identify the molecular weight, we used a regular range protein marker and precision plus protein dual color standard marker. Protein of 10–12 µg was loaded on 4–12% sodium dodecyl sulfate polyacrylamide gel electrophoresis (SDS-PAGE), and then transferred to polyvinylidene difluoride membrane. The membranes were blocked with TBS-T buffer (Tris-buffered saline containing 0.1% Tween 20) containing 5% (w/v) skim milk powder for 1 h at 25°C, and then incubated with primary antibodies for 18 h at 4°C. After incubation with the secondary HRP-linked antibody for 1 h at 25°C, the membranes were detected using Clarity™ Western ECL Blotting Substrates (Bio-Rad, Hercules, CA, USA) and a Bio-Rad imager. Quantification of the images was performed using the Image J software.

In vivo studies. Female ovary-intact athymic nude mice were purchased from Jackson Laboratory at 6 weeks of age. Animals were acclimated for a week upon arrival and then were subcutaneously (s.c.) inoculated in flanks with either MCF-7 cells (1 million cells/animal, 12 animals) or MDA-MB-231 (1 million cells/animal, 18 animals). After the xenograft tumors reached the palpable size of 0.8 cm in diameter, the daily administration of LicA (80 mg/kg.day) or vehicle (4% DMSO, 6% EtOH + 45% water + 45% PEG-400) was started and continued until day 28 after the first dose. Animals were weighed once per week and the tumor size was measured twice per week using a digital caliper. The size of tumors was calculated using the formula [Tumor volume = (A^2) x B/2] with "A" representing the small diameter and "B" representing the large diameter of the tumor. The tumor volume was plotted for each animal every time the tumor was measured. The growth of tumors in animals treated with LicA was compared with growth of tumors in the animals receiving vehicle control. After the completion of the treatments, animals were sacrificed and the tumors were harvested, weighed, and

preserved for future studies. It should be noted that if an animal met any humane endpoint criteria before the completion of the treatment course, as per the IACUC protocol, we sacrificed the animal.

Supplementary Figures.

S1A) Pathways upregulated in microstructures obtained from high-risk women's breast tissue and exposed to LicA ex vivo.

Microstructures from 6 subjects were treated with LicA (5 μM) for 24 h followed by RNA sequencing, differential gene expression, and pathway analysis.

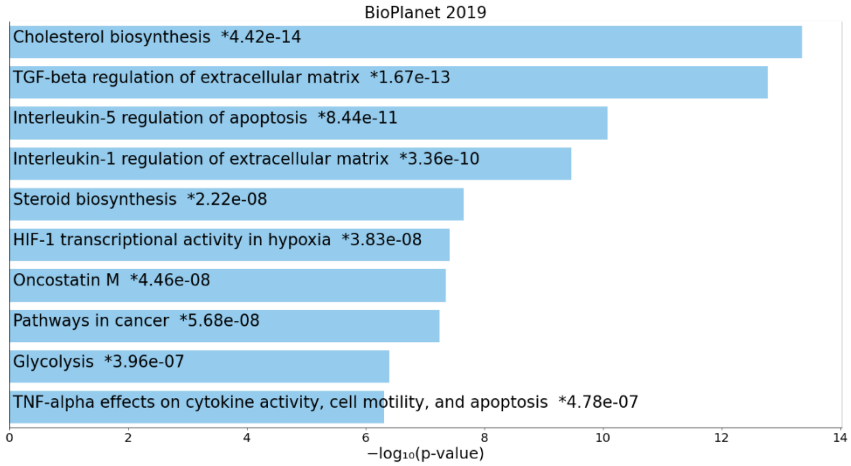

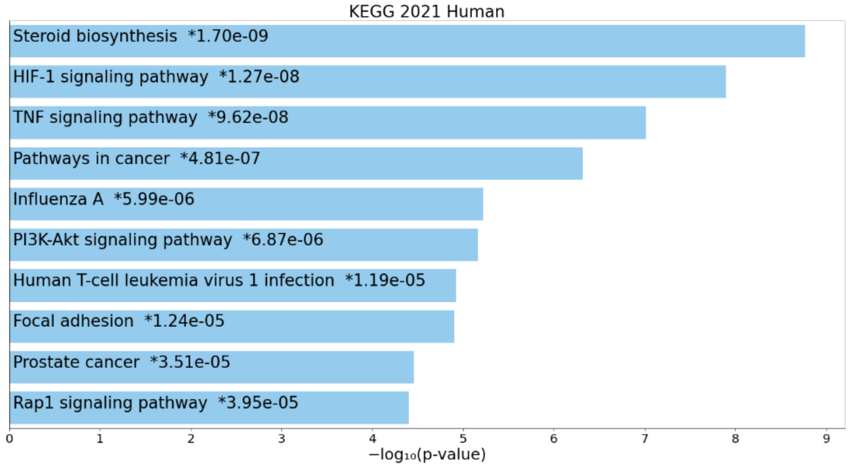

S1B) Pathways downregulated in microstructures obtained from high-risk women's breast tissue and exposed to LicA ex vivo.

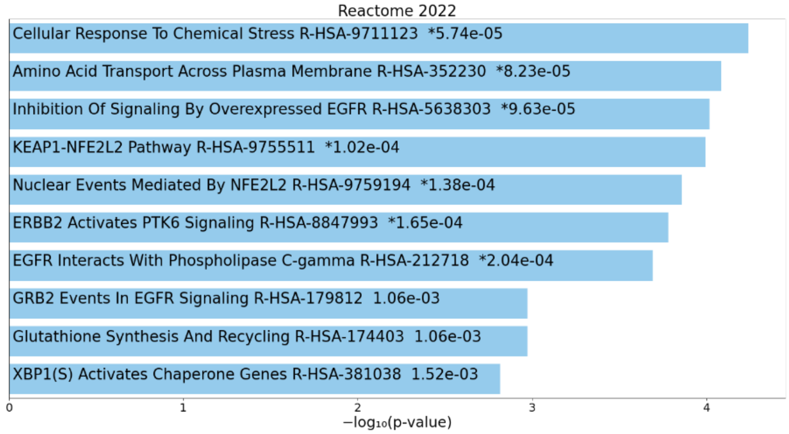

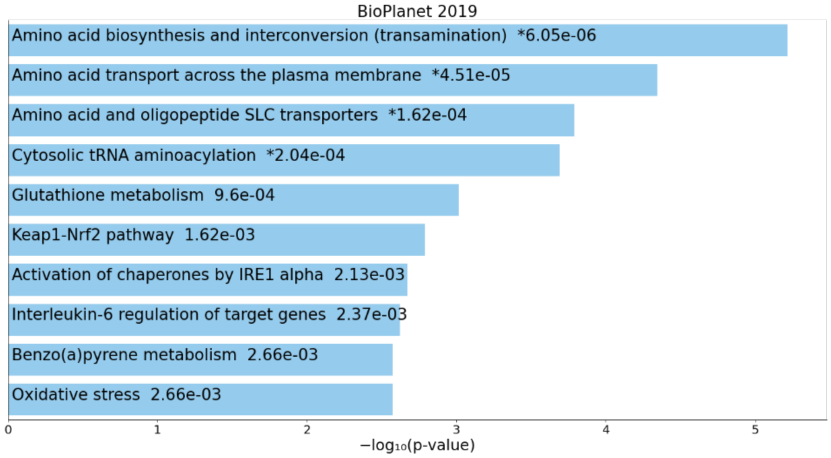

S2) Flux through the cholesterol and steroid biosynthesis as a function of LicA exposure in microstructures obtained from high-risk women's breast.

Microstructures from 6 subjects were treated with LicA (5 µM) for 24 h. RNA sequencing, differential gene expression, and metabolism flux analysis were performed. Orange arrows represent the direction of flux in the treated samples.

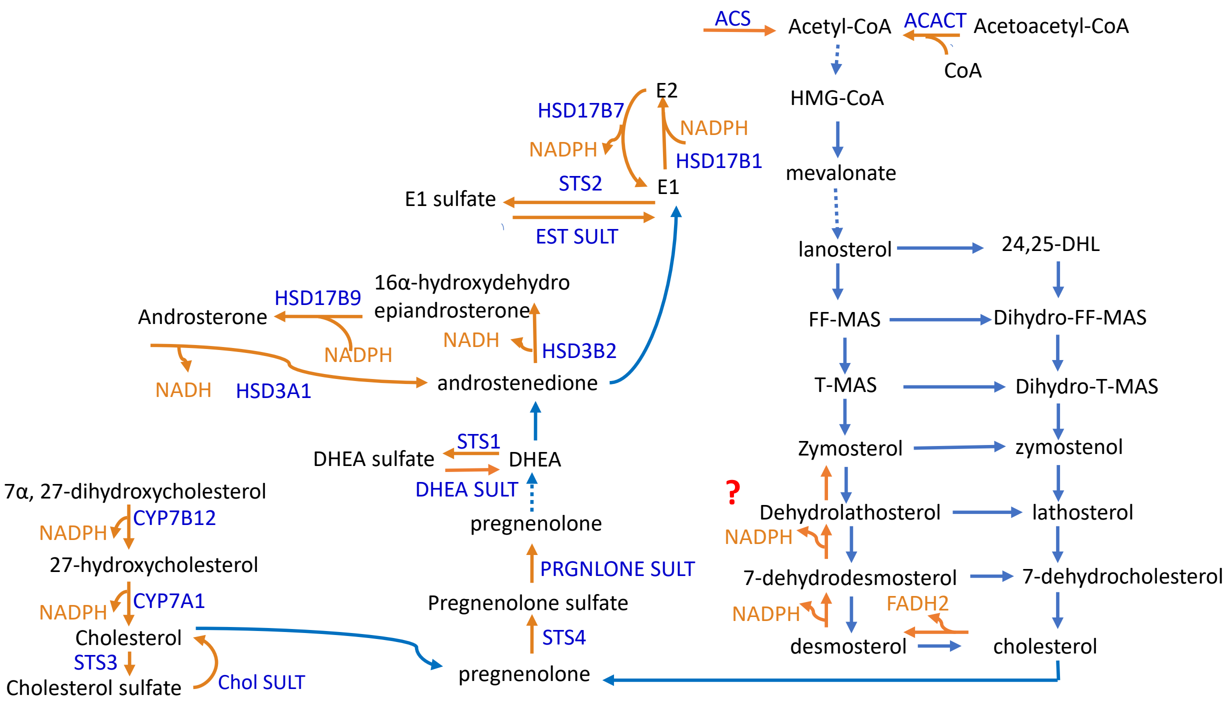

S3A) Heatmap of differentially expressed proteins in MDA-MB-231 cells treated with LicA.

Hierarchical clustering of differentially expressed proteins in MDA-MB-231 cells comparing DMSO-treated control samples (Control_1–3) and LicA-treated samples (Treatment_1–3, 10 μM for 24 h). Cells were lysed, and proteins were analyzed using the PISA proteomics protocol, followed by TMT labeling and LC-MS/MS analysis to capture stabilized and destabilized proteins. The Z-score of protein expression is color-coded, with red indicating upregulation and blue indicating downregulation. Two main protein clusters were identified: Cluster 1 (green) and Cluster 2 (pink). Key pathways associated with differentially expressed proteins are annotated on the left, including Inflammation Response (green), NF-κB Pathway (orange), and Lipid Metabolism (blue). Notable findings include the upregulation of oxidative stress response proteins (GCLC, GCLM, HMOX1, PSPH) in treated samples, along with significant activation of NF-κB-related proteins (SQSTM1, NFKBIE) and lipid metabolism regulators (APOB, PEX19, THBD, CALM1), suggesting a shift toward inflammatory signaling and lipid homeostasis in TNBC cells following LicA treatment.

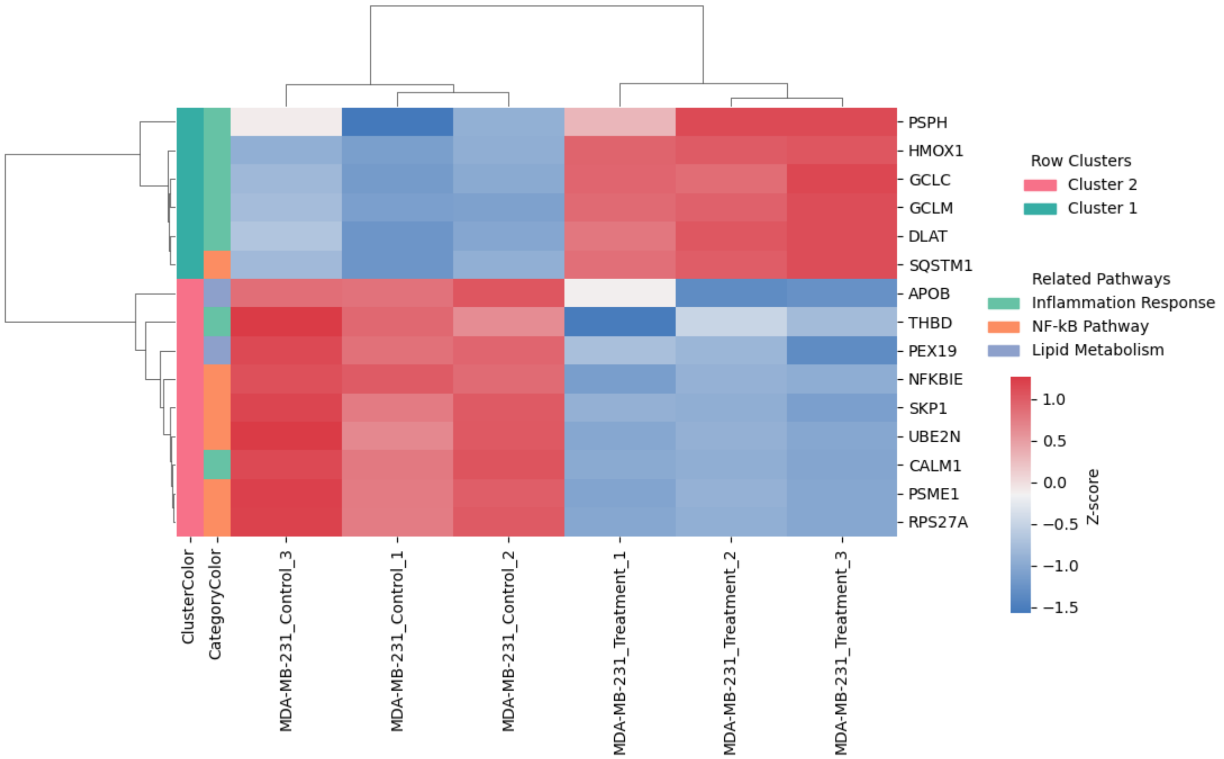

S3B) Heatmap of differentially expressed proteins in MCF-7 cells treated with LicA.

Hierarchical clustering of differentially expressed proteins in MCF-7 cells comparing DMSO-treated control samples (Control_1–3) and LicA-treated samples (Treatment_1–3, 10 μM for 24 h). Cells were lysed, and proteins were processed using the PISA proteomics protocol, followed by TMT labeling and LC-MS/MS analysis to identify stabilized and destabilized proteins. The Z-score of protein expression is color-coded, with red indicating upregulation and blue indicating downregulation. Two main protein clusters were identified: Cluster 1 (green) and Cluster 2 (pink). Pathway annotations highlight proteins associated with Inflammation Response (green) and NF-κB Pathway (orange). LicA treatment resulted in upregulation of oxidative stress-related proteins (GCLC, GCLM, HMOX1, PSPH), mitochondrial metabolism regulators (MT-CO2, PSAT1), and NF-κB-associated immune regulators (TRIM25, IFIH1). Unlike MDA-MB-231, MCF-7 exhibits a mitochondrial metabolic shift rather than NF-κB-driven inflammatory activation, suggesting an alternative adaptation to oxidative stress following LicA treatment.

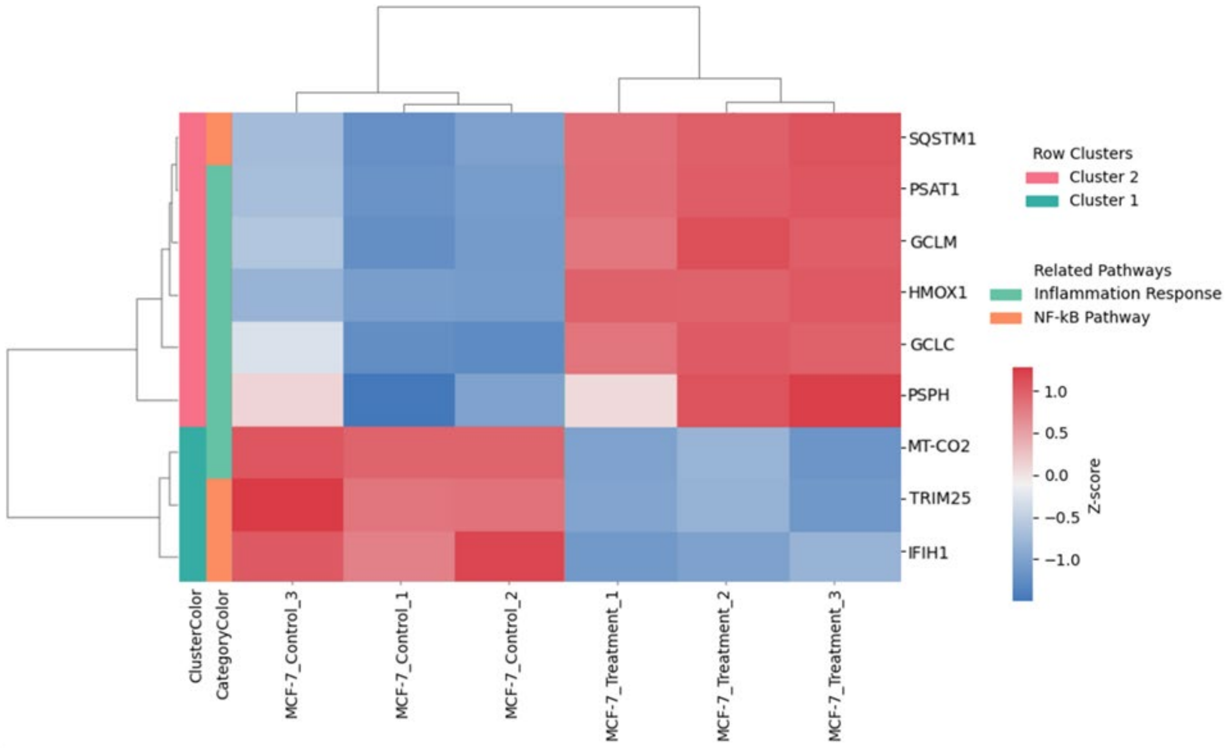

Supplement: Supplementary file 4 — Data S1. Supporting Information. [file IJC-158-1927-s004.pdf]
